# Genome analysis of the foxtail millet pathogen *Sclerospora graminicola* reveals the complex effector repertoire of graminicolous downy mildews

**DOI:** 10.1186/s12864-017-4296-z

**Published:** 2017-11-22

**Authors:** Michie Kobayashi, Yukie Hiraka, Akira Abe, Hiroki Yaegashi, Satoshi Natsume, Hideko Kikuchi, Hiroki Takagi, Hiromasa Saitoh, Joe Win, Sophien Kamoun, Ryohei Terauchi

**Affiliations:** 1 Iwate Biotechnology Research Center, Iwate, Japan; 2 The Sainsbury Laboratory, Norwich, UK; 3 Department of Molecular Microbiology, Tokyo University of Agriculture, Tokyo, Japan; 4 Kyoto University, Kyoto, Japan

**Keywords:** *Sclerospora graminicola*, Graminicolous downy mildew, Oomycetes, Whole genome sequence, Effector, Jacalin-like lectin, *Setalia italica*, Phyllody

## Abstract

**Background:**

Downy mildew, caused by the oomycete pathogen *Sclerospora graminicola*, is an economically important disease of Gramineae crops including foxtail millet (*Setaria italica*). Plants infected with *S. graminicola* are generally stunted and often undergo a transformation of flower organs into leaves (phyllody or witches’ broom), resulting in serious yield loss. To establish the molecular basis of downy mildew disease in foxtail millet, we carried out whole-genome sequencing and an RNA-seq analysis of *S. graminicola*.

**Results:**

Sequence reads were generated from *S. graminicola* using an Illumina sequencing platform and assembled de novo into a draft genome sequence comprising approximately 360 Mbp. Of this sequence, 73% comprised repetitive elements, and a total of 16,736 genes were predicted from the RNA-seq data. The predicted genes included those encoding effector-like proteins with high sequence similarity to those previously identified in other oomycete pathogens. Genes encoding jacalin-like lectin-domain-containing secreted proteins were enriched in *S. graminicola* compared to other oomycetes. Of a total of 1220 genes encoding putative secreted proteins, 91 significantly changed their expression levels during the infection of plant tissues compared to the sporangia and zoospore stages of the *S. graminicola* lifecycle.

**Conclusions:**

We established the draft genome sequence of a downy mildew pathogen that infects Gramineae plants. Based on this sequence and our transcriptome analysis, we generated a catalog of *in planta*-induced candidate effector genes, providing a solid foundation from which to identify the effectors causing phyllody.

**Electronic supplementary material:**

The online version of this article (10.1186/s12864-017-4296-z) contains supplementary material, which is available to authorized users.

## Background

The oomycetes form a diverse group of filamentous eukaryotic microorganisms, also known as water molds, which include saprophytes as well as pathogens of plants, insects, crustaceans, fish, vertebrate animals, and various microorganisms [1, 2]. In plants, pathogenic oomycetes cause devastating diseases in a wide range of species including agricultural crops. Foxtail millet (*Setalia italica* (L.) Beauv.), the second most important millet in terms of global yield [3], suffers from downy mildew disease caused by *Sclerospora graminicola* (Sacc.) Schroet. in regions including India, China, Japan, and Russia.

Twenty genera of downy mildews are known, of which eight are graminicolous downy mildews [4]. Among these, *S. graminicola* (Sacc.) Schroet. is an obligate biotrophic oomycete. The likely source of the *S. graminicola* primary inoculum is oospores remaining in the soil or diseased plant residues. Fourteen graminaceous species are established hosts of *S. graminicola*, with strict host specificity observed among the various isolates of the pathogen [5]. After pathogen invasion, systemically infected leaves generally show chlorosis along the veins. When the pathogen colonizes the branched inflorescences, known as panicles, the floral organs are often transformed into leafy structures, in a process termed phyllody [6]. Phyllody leads to the disease referred to as “witches’ broom”, “green ear disease”, or “crazy top”, and is caused in foxtail millet, pearl millet, maize, and finger millet by pathogens belonging to the three genera, *Peronosclerospora*, *Sclerophthora*, and *Sclerospora* [6, 7]. No induction of phyllody in dicots by downy mildews has been reported.

Whole-genome sequencing and transcriptome analyses have profoundly changed research into plant-microbe interactions in recent years [8], and draft genome sequences of oomycetes have been published for five downy mildew pathogens [9–13]. Whole-genome sequencing has revealed that obligate pathogens including the downy mildews often lose some metabolic pathways, such as for nitrate and sulfate metabolism [9, 10, 13]. In addition, sequence analyses point to conservation of a subset of the effectors that oomycetes secrete to manipulate plant physiology or suppress plant immunity [14]. Such effectors are classified as apoplastic or cytoplasmic based on their localization in the host plants. Apoplastic effectors include (1) secreted hydrolytic enzymes such as proteases, lipases, and glycosylases that can degrade plant tissue, (2) protease inhibitors that protect the oomycetes from host defense enzymes, (3) necrosis and ethylene-inducing peptide 1 (Nep1)-like proteins (NLPs), and (4) PcF-like small cysteine-rich proteins (SCRs) [14]. By contrast, RXLR domain-containing proteins and crinklers (CRNs) are characteristic cytoplasmic effectors in plant pathogenic oomycetes [15, 16]. Several genomic sequences for oomycetes and dicot downy mildews have been released; however, with the exception of the recently published transcriptome analysis of pearl millet infected with *S. graminicola* [17], there have been no genomic analyses of the graminicolous downy mildew pathogens.

Here, we perform whole-genome sequencing on the *S. graminicola* strain that infects foxtail millet. We further report RNA-seq–based gene prediction and annotation of the *S. graminicola* genome, and expression profiling of the putative secreted protein genes flagged as effector candidate genes.

## Results

### De novo assembly of the *S. graminicola* (*Sg*) genome

We prepared genomic DNA from a mixture of sporangia and zoospores colonized on the leaves of foxtail millet. The genome was sequenced using an Illumina platform and a paired-end library with a mean insert size of 370 bp, as well as mate-pair libraries with insert sizes of 2, 4, and 6 kbp. To check for contamination with bacterial and host plant DNA, some of the short reads were assembled using Platanus v.1.2.1 [18], and the generated contigs were used for a BLASTn search against the NCBI nt database. Of the 97 scaffolds over 200 bp in length, 11 scaffolds showed a high similarity to other oomycete or fungal sequences (Additional file 1: Table S1). The others did not show any significant similarity to the sequences in the database. From this result, we judged that the level of contamination from bacterial and host plant DNA was negligible, and proceeded to de novo assemble all the sequencing reads that had sufficient Phred quality scores.

**Fig. 1:**
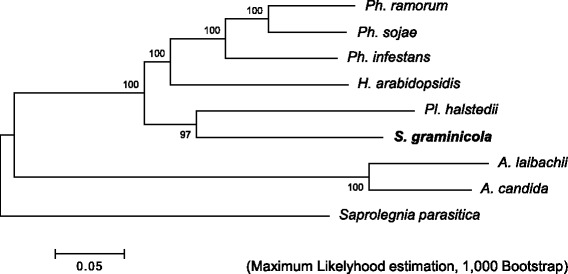
Phylogenetic relationship of oomycete genomes. The tree was generated based on the nucleotide sequences of orthologous genes predicted by CEGMA pipeline using the Maximum Likelihood method implemented in MEGA6.06-mac. Bootstrap values from 1000 replicates are indicated on the branches

The filtered Illumina sequencing reads were used for the de novo assembly in Platanus v.1.2.1 (Table 1). The total size of the assembled contigs was 254 Mbp, with an N50 scaffold length of 24.3 kbp. The longest contig was 279 kbp. The completeness of the assembled genome was analyzed using the CEGMA pipeline [19]. Complete and partial mapping identified 95.56% and 98.39% of the 248 core eukaryotic genes (CEGs) in the *Sg* sequence, respectively, suggesting that our *Sg* draft genome sequence was of sufficient quality for further analysis and gene prediction. Phylogenetic analysis using the CEGs of available oomycete genomes revealed that *Sg* is closely related to *Plasmopara halstedii* (*Plh*), which infects sunflower (Fig. 1).

**Table 1:** Genome statistics of *Sclerospora graminicola* (*Sg*) and other previously sequenced oomycetes^a^

Characteristic	*Sg*	*Plh*	*Hpa*	*Phi*	*Phs*
Estimated genome size	360 Mbp	100 Mbp	100 Mbp	240 Mbp	95 Mbp
Number of scaffolds	64,505	3162	3408	4921	1810
N50 scaffold length	24.3 kbp	1540 kbp	332 kbp	1570 kbp	463 kbp
Total scaffold length	254 Mbp	75 Mbp	78.4 Mbp	228.5 Mbp	86.0 Mbp
GC content	46.4%	45.3%	47%	51.0%	54.4%
Repeat (%)	73%	40%	42%	74%	39%
Number of genes	16,736	15,469	14,321	17,787	18,969
Secreted protein genes	1220	631	762	1568	1701
CEGMA					
Group 1^b^	92.42%	93.94%	89.5%	96.97%	96.97%
Group 2 ^b^	94.64%	96.43%	96.5%	96.43%	98.21%
Group 3 ^b^	98.36%	98.36%	98.5%	96.72%	100.00%
Group 4 ^b^	96.92%	100.00%	97.0%	96.92%	98.46%

^a^ *Plh Plasmopara halstedii*, *Hpa Hyaloperonospora arabidopsidis*, *Phi Phytophthora infestans*, *Phs Phytophthora sojae*

^b^The CEGs are split into 4 groups with Group 1 being the least conserved between organisms, and Group 4 being the most conserved between organisms

### *Sg* has a large and heterozygous genome

**Fig. 2:**
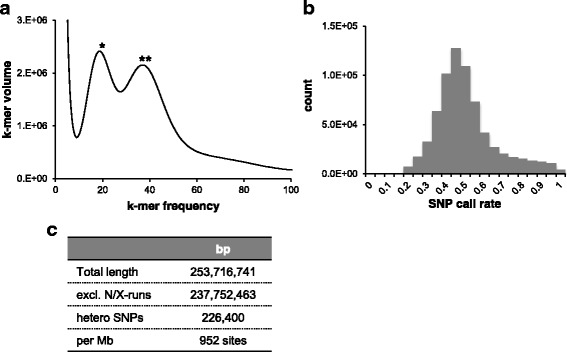
*Sg* has a large and diploid genome with high heterozygosity. **a** K-mer distribution and coverage of sequencing reads at K = 15. Peaks with single and double asterisks were estimated as k-mer species derived from heterozygous (k-mer frequency = 19) and homozygous (k-mer frequency = 37) sequences, respectively. **b** Ploidy analysis displaying the distribution of the SNP call rate. **c** Heterozygosity was evaluated by counting the SNPs based on the alignment of genome sequence reads

Analysis of the k-mer frequency using paired-end reads showed two peaks, possibly derived from heterozygous and homozygous DNA sequences (Fig. 2a). To estimate the ploidy level of the *Sg* genome, we analyzed the distribution of the biallelic SNP call rate (Fig. 2b). The SNP counts had a single mode around 0.5, suggesting that the genome was diploid. The number of heterozygous SNPs, with a call rate of between 0.4 to 0.6, was 226,400 (Fig. 2c). The total genome size, estimated from the k-mer frequency at the peak corresponding to the putative homozygous DNA, was approximately 360 Mbp.

### *Sg* has a highly repetitive genome

Gene prediction was carried out using Trinity/PASA, Tophat2/Cufflinks/PASA, MAKER2, AAT, based on the RNA-seq data and the in silico method [20–25]. By combining multiple types of evidence using EvidenceModeler [22], we identified a total of 16,736 genes supported by RNA-seq data. Analysis of repeated sequences using RepeatModeler [26] and RepeatMasker [27] revealed that approximately 73% of the assembled genome was repetitive, with more than half composed of long terminal repeat (LTR)-elements (Additional file 2: Table S2).

### The *Sg* genome encodes proteins comparable to those of other downy mildews

To compare the *Sg* genome with those of other oomycetes, we performed clustering analyses of orthologs and paralogs from three downy mildew pathogens (DMs) (*Sg*, *Plh*, and *Hyaloperonospora arabidopsidis*; *Hpa*) and two *Phytophthora* species (*Ph. infestans*; *Phi* and *Ph. sojae*; *Phs*) based on the OMA orthology database [28]. There were 3548 and 2725 common orthologous groups in the DMs and in the five genomes (three DMs plus the two *Phytophthora* species), respectively (Additional file 3: Table S3). A total of 2055 groups were conserved in the *Phytophthora* species but not in the DMs, while only 128 groups were conserved among the DMs but not in *Phytophthora*. Some obligate biotrophs have lost the nitrogen and sulfate metabolic pathways [9, 10, 13]; an ortholog search revealed that *Sg* similarly lacked nitrate reductase, nitrite reductase, nitrate transporter, glutamine synthetase, and cysteine synthetase (Additional file 4: Table S4).

To gain insights into the unique features of the *Sg* genome, we compared the frequency of the protein domains encoded in the five oomycete genomes. In *Sg*, 11 domains were overrepresented (Fisher’s exact test, *p* < 0.05), compared with two in the DMs and/or *Phytophthora* species (Additional file 5). In particular, the Jacalin-like lectin domain was overrepresented among the putative secreted proteins. Although no domains were underrepresented in *Sg* alone, 85 domains were underrepresented in the three DMs in comparison with the *Phytophthora* species. Of these 85 domains, 20 were associated with cellular transporters and 11 were linked to plant cell wall degradation. Several protein families related to plant defense, such as elicitin and cellulose-binding elicitor lectin, were also less common in the DM genomes than in *Phytophthora* (Additional file 5).

### *Sg* expresses conserved effector-like protein genes during infection

A total of 1220 *Sg* proteins were classified as putative secreted proteins based on the presence of signal peptides, predicted by SignalP4.1 [29], and the absence of transmembrane domains. This total was greater than those of *Plh* and *Hpa*, but fewer than that of *Phi* (Table 1). The number of proteins related to pathogenicity in *Sg* was comparable to that in other DMs, except for the RXLR-like proteins, of which *Sg* had more than *Plh* but fewer than those in the *Phytophthora* species (Table 2).

**Table 2:** Summary of putative pathogenicity genes in *Sclerospora graminicola* and related oomycetes

Genes encoding	*Sg*	*Plh*	*Hpa*	*Phi*	*Phs*
Serine protease ^a^	32 (5)	30	28	34	31
Aspartic protease ^a^	6 (1)	5	5	6	6
Cysteine protease	14 (4)	15	16	18	17
Metalloprotease ^a^	26 (3)	30	30	32	29
Kazal-like serine protease inhibitor ^b^	10 (9)	16	5	34	23
Cystatin-like cysteine protease inhibitor ^b^	1 (1)	2	0	3	2
Cutinase ^b^	2 (2)	2	2	4	16
Pectate lyase ^b^	8 (5)	3	12	46	46
Pectin lyase ^b^	11 (7)	5	4	11	19
CAP domain ^b, e^	20 (12)	22	15	30	40
NPP1-like ^b^	24 (17)	19	21	27	74
Elicitin-like ^b^	17 (10)	16	16	44	56
RXLR-like ^c^	355 (355)	274 ^d^	134 ^d^	563 ^d^	396 ^d^
CRN-like	45 (4)	77 ^d^	20 ^d^	196 ^d^	100 ^d^

^a^PANTHER11.0 classification database, ^b^: Interproscan, ^c^: presence of N-terminal putative secretion signal and RXLR motif. ^d^: reported in previous papers. ^e^: CAP domain indicates Cysteine-rich secretory protein. Numbers in parentheses indicate numbers of putative secreted protein genes

To search for effector candidates involved in *Sg* infection, an RNA-seq analysis was performed using total RNA extracted from sporangia/zoospores (inocula) and infected leaves. The foxtail millet leaves were inoculated with a spray containing a mixture of sporangia and zoospores. Primary penetration hyphae appeared 16–18 h after inoculation, and haustoria were formed one day after inoculation. We analyzed the gene expression profiles at five time points (stage 1: SPO (sporangia and zoospores), stages 2, 3, 4, and 5: 16 hpi (hpi; hours post infection), and 1, 2, and 3 dpi (dpi; days post infection), respectively). Distribution of maximum transcripts per million (TPM) value of all genes in five data points indicated that 54% of the genes were lower than 20, 31% were from 20 to 100, 14% were from 100 to 1000, and 1.6% were higher than 1000. From differentially expression gene (DEG) analysis using edgeR [30], expression of 91 putative secreted protein genes significantly changed during infection. The maximum value of TPM of all DEGs was more than 20.

**Fig. 3:**
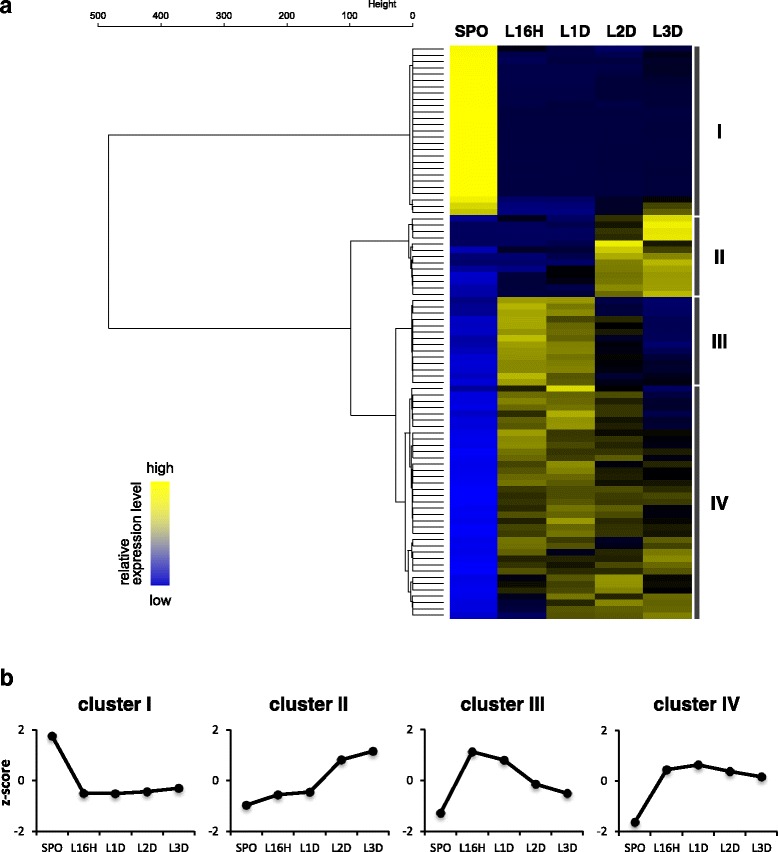
Transcriptome profile of *Sclerospora graminicola* infection. **a** Heat map showing the expression patterns of DEGs encoding putative secreted proteins. **b** Line plots of the expression patterns of each gene cluster. SPO: mixture of sporangia and zoospores; L16H: SPO-inoculated leaves 16 h after inoculation; L1D, L2D, and L3D: SPO-inoculated leaves at one, two, and three days after inoculation, respectively

Ninety one DEGs were classified into four clusters based on their expression patterns using ward’s method (Fig. 3, Additional file 6: Table S5). Representative genes of each cluster were validated by quantitative reverse transcription PCR (qRT-PCR) (Additional file 7: Fig. S1). Cluster I included genes expressed in sporangia or zoospores, but not during infection. The expression of genes belonging to cluster II increased in late stage of infection, suggesting that they include components contributing to pathogen expansion into leaves and the absorption of nutrition from host cells. Genes belonging to clusters III and IV were induced during stage 2 when the primary penetration hyphae developed, after which the expression of genes in clusters III gradually returned to basal levels. To determine the gene families overrepresented in each cluster, an enrichment analysis of protein domains predicted by InterProScan was performed (Additional file 8). CAP domain (CAP: the cysteine-rich secretory proteins, antigen 5, and pathogenesis-related 1 proteins superfamily proteins) and CUB domain which is related to Trypsin-like peptidase were enriched in cluster I. Jacalin-like lectin domain and Necrosis inducing protein domain were significantly enriched in cluster III, indicating that these domain could function in the early stages of *Sg* infection.

Different clustering methods could provide different results. We additionally performed clustering analyses using two methods, logFC-Cosine method using the cosine similarity of the vectors of their log-fold-change (logFC) values (Additional file 9: Figure S2) and model-based clustering method [31] (MBCluster; Additional file 10: Figure S3). Cluster I was separated into two clusters and some genes of cluster III and IV were classified into the same cluster by logFC-Cosine and MBCluster, however, most of genes showed similar clustering patterns by multiple clustering methods (Additional file 6: Table S5). Interproscan domain enrichment analysis indicated that Jacalin-like lectin domain and Necrosis inducing protein domain were also enriched in cluster 4 of logFC-Cosine method and cluster 2 of MBCluster that contain genes induced in early infection phase (Additional file 8).

To reveal features of *Sg* secretome, putative secreted proteins of *Sg* and 11 oomycetes (*Plh*, *Hpa*, *Phi*, *Phs*, *Ph. ramorum*, *Ph. capsici*, *Ph. parasitica*, *Albugo candida*, *A. laibachii*, *Pythium ultimum*, *Saprolegnia parasitica*) were clustered using TribeMCL protein family clustering algorithm [32]. 13,328 proteins were clustered into 1252 families (each family contains at least two sequences) and 1862 singletons. Of the 1252 familes, 230 contained *Sg* and other oomycete proteins and 78 were *Sg* specific families. *Sg*-specific families consisted of 39 RXLR-like families, 4 Jacalin-like domain-containing protein families, one leucine-rich repeat domain-containing family, one Mitochondrial carrier domain-containing family, and 33 unknown protein families (Additional file 11). Of these *Sg*-specific Tribes, Jacalin-like domain-containing families included genes those have high TPM levels, especially in stage 2 and 3 (Additional file 12: Fig. S4).

### Jacalin-like lectin domain proteins

**Fig. 4:**
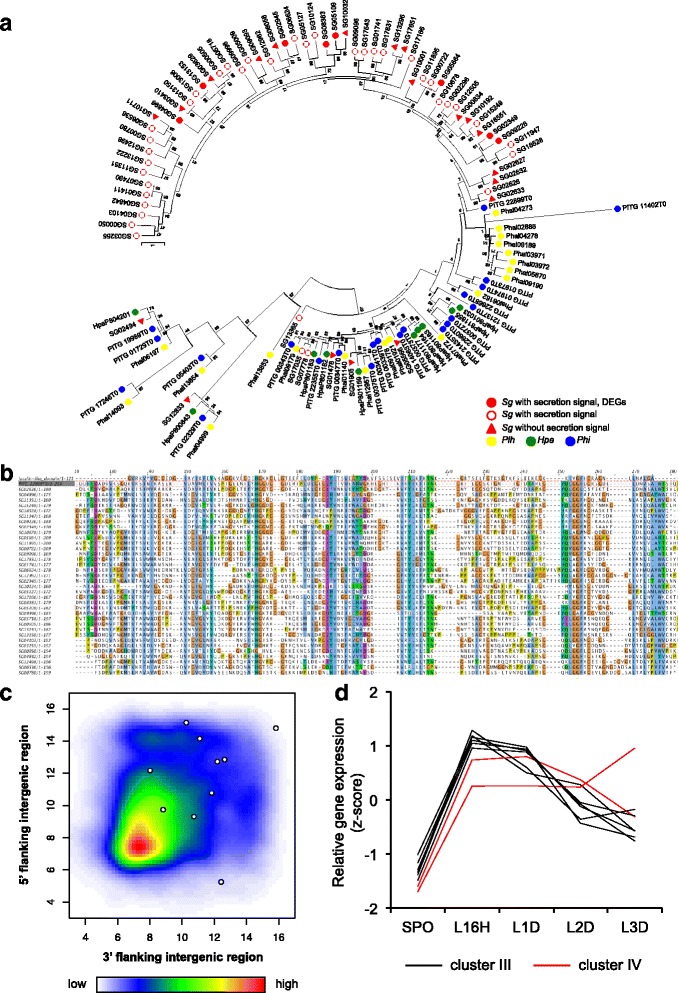
Features of jacalin-like lectin domain-containing protein genes. **a** Phylogeny of the jacalin-like lectin domain-containing proteins of *Sg*, *Plh*, *Hpa*, and *Phi*. The tree was conducted using the Maximum Likelihood method implemented in MEGA6.06-mac, with 1000 bootstrap replicates. **b** Multiple sequence alignment showing the sequence similarity between PITG22899T0 and the jacalin-like lectin domains of the Sg proteins. **c** Distribution of intergenic region length of Sg genes. All predicted genes are represented by a heatmap and the jacalin-like protein genes are represented by white circles. **d** Relative expression of DEGs of jacalin-like protein during infection. Clusters III and IV are defined in Fig. 3

Jacalin-like lectin domain-containing proteins belong to a subgroup of lectins with binding specificity to mannose or galactose, and are involved in multiple biological processes. Jacalin-like proteins were overrepresented in the *Sg* genome (Additional file 5), and a phylogenetic analysis indicated many were specific to *Sg* (Fig. 4a). Among the jacalin-like protein genes of *Plh*, *Hpa*, and *Phi*, the closest to the *Sg*-specific clade was PITG_22899. Intriguingly, most of the *Sg*-jacalin-like proteins, including proteins with putative secreted signals and significant expression levels, belonged to the *Sg*-specific clade (Fig. 4a, red filled circles, Additional file 13). Effector genes are distributed in gene-sparse regions of the *Phi* genome [33, 34]. From the analysis of intergenic distance, jacalin-like protein genes appeared to distribute in gene-sparse regions (Fig. 4c, Wilcoxon rank sum test, 5′-intergenic length; *p*-value = 0.03721, 3′-intergenic length; *p*-value = 0.01161), however, most of jacalin-like protein genes were located near the scaffold border and were not possible to determine intergenic distance.

### Nep1-like proteins (NLPs)

NLPs are a widespread effector family among filamentous and bacterial pathogens that show very different lifestyles [35]. Oomycetes have two types of NLPs: type 1 NLPs with a cation-binding pocket required for cytotoxicity, and type 1a NLPs with amino acid substitutions in their cation-binding pocket [35]. The *Sg* genome contained 24 NLP-encoding genes, 17 of which had an N-terminal secretion signal peptide (Additional file 14). One NLP, SG00816, was classified as a type 1 NLP with a TRAP repeat and the other 23 were type 1a NLPs.

**Fig. 5:**
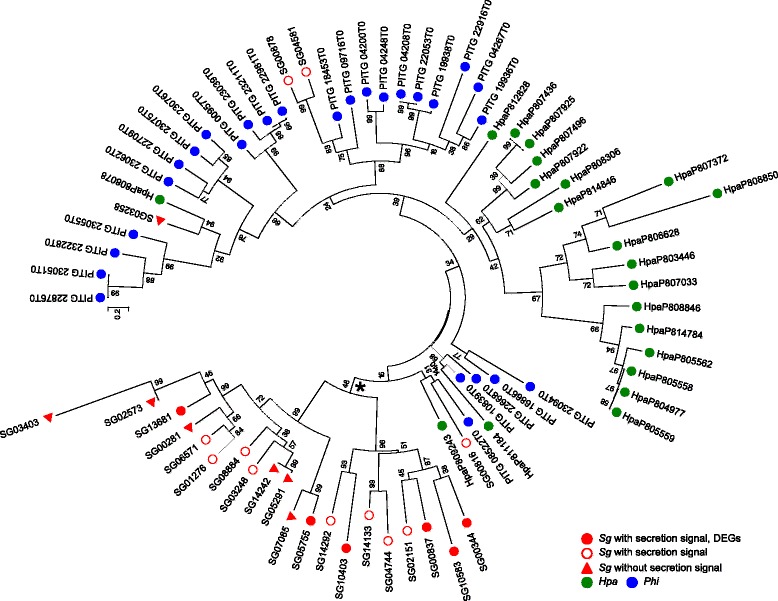
Phylogenetic relationship of *NLP* genes in *Sg*, *Hpa*, and *Phi*. The tree was constructed using the Maximum Likelihood method implemented in MEGA6.06-mac, with 1000 bootstrap replicates. The asterisk indicates the *Sg*-specific expansion group

Six of the 24 *SgNLP*s were DEGs (Additional file 14). The type 1 NLP, SG00816, was not significantly expressed at any stage (Additional file 14). Intriguingly, these DEGs of NLPs were in one clade of the *Sg*-specific expansion groups (see asterisk in Fig. 5). All of six differentially expressed NLPs were classified into cluster III and IV (Additional file 14).

### Crinklers (CRNs)

CRNs are cytoplasmic effectors originally identified in *Phi* as secreted proteins that have a conserved LFLAK motif in the 50 amino acid residues of the N-terminal [36]. We identified 45 CRN-like genes in *Sg* (Table 2). Only four of these had a signal peptide at the N-terminus. *SgCRN*s, including four putative secreted *CRN* genes, were not significantly expressed during infection (Additional file 15).

### RXLR-like proteins

**Fig. 6:**
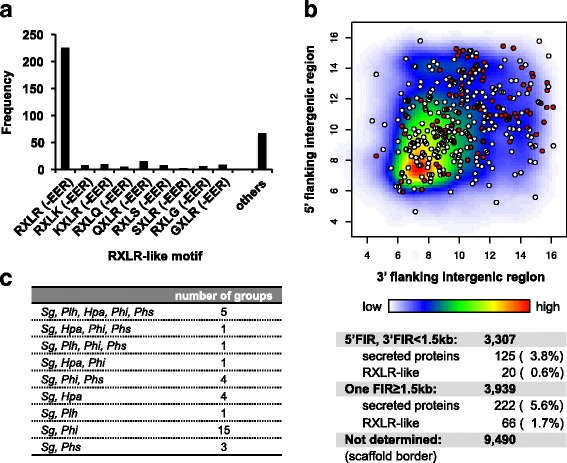
Features of RXLR-like protein genes. **a** Distribution of the conserved sequence patterns of putative RXLR-like proteins. **b** Distribution of *Sg* genes according to the length of their 5′ and 3′ flanking intergenic regions. The density of genes in each positional bin is indicated by a heatmap. Putative secreted proteins (white) and RXLR-like proteins (red) genes are represented by circles. (C) Orthologous groups of SgRXLR-like proteins within the putative secreted proteins of four oomycetes

The RXLR domain is a putative host-targeting motif [37] and is highly conserved among plant-pathogenic oomycetes. We predicted RXLR-like protein genes by searching for a RXLR(−EER) sequence following the N-terminal putative signal peptide. Proteins showing high similarity to known RXLR-like proteins were also included as RXLR-like protein candidates. A total of 355 RXLR-like proteins were found, among which 165 had the exact RXLR-EER motif and 60 had the RXLR motif, while 130 were predicted to be RXLR(−EER) variants (Fig. 6a). Some RXLR effectors contain a core α-helical fold known as the WY-fold [38]. We explored whether our identified RXLR-like proteins had the WY-fold using HMMER, and found a total of 38 proteins with at least one WY-fold (Additional file 16). In the gene expression profile and expression pattern clustering, RXLR-like protein genes were not enriched in any clusters; however, 22 of these genes were induced during infection (Additional files 8 and 15).

Effector genes are distributed in gene-sparse regions of the *Phi* genome [33, 34]. In the *Sg* genome, secreted protein genes, in particular RXLR-like protein genes, were distributed in relatively gene-sparse regions compared with all of the predicted genes (Fig. 6b). Wilcoxon rank sum test indicated that distribution of intergenic length of RXLR-like genes was significantly different from that of all predicted genes (5′-intergenic length; *p*-value = 9.244e-05, 3′-intergenic length; *p*-value = 1.225e-08). We searched for orthologs of SgRXLR-like proteins among the putative secreted proteins of five oomycetes (*Sg*, *Plh*, *Hpa*, *Phi*, and *Phs*) and compared them using the OMA orthology database [28]. There were 35 ortholog groups that contained SgRXLR-like proteins (Fig. 6c), with most *Sg* orthologs found in the *Phi* genome.

## Discussion

### *S. graminicola* (*Sg*) has a large and highly heterozygous genome

Our analysis of Illumina sequencing paired-end reads suggested that the genome size of *Sg* is approximately 360 Mbp. This is 1.3 times larger than the genome of *Phytophthora mirabilis*, the largest among the previously sequenced oomycete plant pathogen genomes [39]. Phylogenetic analyses indicated that *Sg* is closely related to *Plh*, which has a 100-Mbp genome (Table 1), suggesting that expansion of the *Sg* genome probably occurred after its divergence from *Plh*. A broad range of genome sizes among closely related oomycetes is also found in *Phytophthora*; the smallest genome among the deeply sequenced *Phytophthora* species is 65 Mbp (in *Ph. ramorum*), while the largest genome is 240 Mbp (in *Ph. infestans*) [33]. Genome expansion occurred in *Ph. infestans* with an increase in repetitive regions such as the Gypsy elements. We found that at least 73% of *Sg* and 40% of *Plh* genomes comprised repeat regions, respectively. The number of protein-coding genes in the *Sg* genome was comparable to that in *Plh,* indicating that the larger genome size in *Sg* is not caused by an increased number of genes but by the expansion of the repetitive elements.

### Proteins encoded by the *Sg* genome are mostly comparable to those of dicot downy mildews

A total of 2055 orthologous gene groups were conserved in the *Phytophthora* species but not the DMs. By contrast, the number of groups conserved among the DMs but not in *Phytophthora* was only 128. This suggests two possibilities: either the *Phytophthora* species are more phylogenetically closely related while the DMs are more diversified, or the obligate biotrophs have lost substantial numbers of genes in comparison with non-obligate microbes. Indeed, the DMs, including *Sg*, lack part of the nitrogen and sulfate metabolic pathways. When we compared the protein-coding domain frequency between the DM and *Phytophthora* genomes, we found fewer genes encoding transporters, cell wall degrading enzymes, and elicitin in the DMs than in *Phytophthora*. These results suggest that DMs have adapted to their hosts and developed their obligate biotroph lifestyles by losing components that might induce the host defense response.

### Expression patterns of putative secreted protein genes

We performed expression profiling of putative secreted protein genes during infection and classified them into five clusters. Cluster I included genes expressed only in sporangia and zoospores, which likely having no direct influence on *Sg* infection of foxtail millet leaves. By contrast, the expression of genes belonging to clusters II, III, and IV increased during *Sg* infection in foxtail millet leaves. Genes of cluster II gradually increased with development of internal hyphae, suggesting that these genes contribute to the haustorial development of *Sg* and might be involved in the induction of phyllody in the *Sg*-infected foxtail millet plants. The expression of genes belonging to clusters III and IV were induced in stage 2 of infection, during the development of the primary penetration hyphae, then subsequently returned gradually to their basal expression levels. We hypothesize that *Sg* genes belonging to these clusters have roles in overcoming the host defense responses in foxtail millet, and that the effector candidate genes determining host specificity are included in clusters II, III, and IV.

### Jacalin-like lectin domain proteins

We found that jacalin-like lectin domain-containing protein genes were specifically overrepresented in the *Sg* genome in comparison with *Plh*, *Hpa*, *Phi*, and *Phs* (Additional file 5). Additionally, clustering of *Sg* and 11 oomycetes secretomes using TribeMCL showed that *Sg* has four *Sg*-specific families which include 36 genes of jacalin-like domain proteins. PITG_22899, the closest gene to the *Sg*-specific clade, is induced in *Phi* during plant infection stages*,* and has been reported as an effector candidate by an in silico analysis (Fig. 4a) [34]. These findings imply that jacalin-like genes play a role in infection and have specifically diversified in the *Sg* genome. Our clustering analysis of the *Sg* gene expression patterns indicated that eight jacalin-like protein genes were found as DEGs (Additional file 13, in cluster III and IV). Many of jacalin-like genes other than DEGs also indicated high level of TPM (Additional file 13) implying that jacalin-like genes play roles during early infection.

If jacalin-like proteins are a novel class of effectors in *Sg*, it would be reasonable to expect the jacalin-like genes to be distributed in gene-sparse regions. While this did appear to be the case (Fig. 4c) [33, 34], the assembled scaffolds in this study were too short to determine genetic distances for a large number of genes. The use of long sequencing reads to improve the assembly will be required to determine the genetic distances of all genes, in particular the effector candidates located in gene-sparse regions.

Previous reports suggested that plant jacalin-like proteins play a role in the defense response; for example, a jacalin-related lectin-like gene in wheat positively regulates resistance to fungal pathogens [40]. The authors reported that Ta-JA1 and OsJAC1 function in bacterial and fungal resistance in wheat and rice plants, respectively. Both proteins belong to a Poaceae-specific protein family, the members of which contain jacalin-related lectin and dirigent domains [41, 42]. Analysis of the separated domains of OsJAC1 indicated that the jacalin-related lectin domain is important for its targeting to the site of pathogen attack [42]. Another study revealed that six of eight grass species have nucleotide-binding leucine-rich repeat (NLR) protein genes including jacalin domain-encoding sequences [43]. These reports imply that jacalin-like lectin domains play a role in defense responses in the Gramineae plants. The foxtail millet genome contained one NLR-jacalin fusion protein gene and four jacalin-like protein genes with a dirigent domain (Additional file 17). Taken together, the previous reports and the results of the present study suggest the possibility that *Sg* secretes jacalin-like proteins to disturb host immune signaling, enabling it to successfully establish an infection. Future studies should determine the function of *Sg* effectors containing the jacalin-like lectin domain.

### Nep1-like proteins (NLPs)

Oomycetes have cytotoxic-type (type 1) and non-cytotoxic-type (type 1a) NLPs. [35]. In *Hpa* and *Plh*, most NLPs were classified as type 1a [9, 13]. Although the *Sg* genome contained 24 NLP-encoding genes, only one, SG00816, was a type 1 NLP. The expression of SG00816 was very low, suggesting that this protein plays only a minor role in infection (Additional file 14). In hemibiotrophs such as *Phytophthora* and *Colletotrichum higginsianum*, cytotoxic NLPs are believed to control the transition from the biotrophic phase to the necrotrophic phase by inducing cell death in the host plants [44, 45]. DMs are biotrophic pathogens, and cytotoxic NLPs are presumably not required for their lifecycles.

Non-cytotoxic NLPs are expressed in the biotrophic phase of hemibiotrophic pathogens [45–48]; therefore, they are believed to play a role in host penetration or the establishment of infection [35]. Intriguingly, expression of 12 *SgNLPs* including six DEGs peeked at 16 hpi (Additional file 14). These results suggest that the SgNLPs also play a role in the establishment of *Sg* infection in foxtail millet.

### CRNs and RXLR proteins

Oomycetes have cytoplasmic effectors belonging to the RXLR and CRN protein families, which comprise many members [9–13]. A total of four CRNs and 355 RXLR protein genes with putative secreted signals were predicted in the *Sg* genome. In addition to four CRNs in putative secrete proteins, there are 41 CRN-like proteins without N-terminal secretion signals (Additional file 15), in agreement with a previous report that a large number of non-secreted CRNs are present in the *Plh* genome [13]. Our RNA-seq analysis revealed that 21 RXLR protein genes were found as DEGs during infection, and could have roles as effectors in *Sg*. Clustering analysis based on protein sequence using TribeMCL indicated that there are 39 RXLR families belonging to the *Sg*-specific tribes. However, expression patterns were not similar among RXLR-like genes and RXLR-like genes were not enriched in any clusters like as jacalin-like genes (Additional file 8). These results suggest that roles of RXLRs are not correlated with sequence similarities. By contrast, the expression levels of the four CRN genes were very low, suggesting that they may only have minor roles in *Sg*-foxtail millet interactions. Of the 355 RXLRs, 165 had the exact RXLR-EER motif. This contrasted with the situation in *Plh,* the most closely phylogenetically related oomycete to *Sg*, in which only 34 of 274 RXLRs had a typical RXLR-EER motif [13]. An ortholog search indicated low numbers of orthologs among the related oomycetes (Fig. 6c). Considering the above findings, the RXLRs may have evolved separately in each species, depending on the process of interaction with their host plants.

## Conclusions

In this study, we report the first genome sequence of a graminicolous downy mildew pathogen, *S. graminicola.* Although the relatively large *Sg* genome showed high heterozygosity and was repetitive, it encoded a similar number of genes to other oomycete genomes. A phylogenetic analysis indicated that *Sg* was most closely related to *Plh* among the oomycetes for which genome sequences are available; however, the significantly smaller genome of *Plh* suggested that the genome expansion of *Sg* occurred after its divergence from *Plh*. Gene prediction and transcriptome analysis revealed that the *Sg* genome had several of the common effectors conserved throughout the oomycetes. In addition, *Sg* had a species-specific clade of jacalin-like lectin protein genes that were distributed in gene-sparse regions of the genome. Further analyses are needed to address the function of these jacalin-like genes and to determine whether other graminicolous downy mildews have homologous jacalins. The resources provided in this study will be invaluable for future advances in understanding the pathology of *S. graminicola*, and to determine how this pathogen perturbs host development.

## Methods

### Plant and oomycete materials

The foxtail millet (*Setaria italica* (L.) P. Beauv*,* cultivar ‘Ootsuchi-10’), obtained from the experimental field of Iwate Agricultural Research Center (IARC), Karumai, Iwate, Japan with a permission, was used in this study. The single zoospore isolated strain of *Sclerospora graminicola* (Sacc.) Schroet. was derived from the isolate collected in the IARC field with a permission in 2013. Plants were grown in an artificial climate chamber at 20–25 °C with 15 h light. Four-week-old plants were infected with *S. graminicola* by spraying them with a mixture of sporangia and zoospores (1–5 × 10^5^ per mL). Seven days after inoculation, the leaves were harvested, incubated in 70% ethanol for 30 s, rinsed with distilled water, and used for inoculum preparation. Sporulation was induced by incubating the infected leaves at 100% humidity at 20 °C for 5–6 h. Mixtures of sporangia and zoospores were collected by rinsing the sporulated leaves with chilled sterile water.

### DNA extraction

Genomic DNA was prepared from spores. The spores were ground in liquid nitrogen, to which CTAB buffer (140 mM sorbitol, Tris-HCl (pH 8.0), 22 mM Na-EDTA, 800 mM NaCl, 1% sarkosyl, and 0.8% CTAB (hexadecyltrimethylammonium bromide)) was added, before being mixed and incubated at 65 °C for 10 min. The lysate was then mixed with chloroform and centrifuged at 20,000×*g* for 5 min, after which the upper phase was transferred and precipitated using isopropanol. The DNA pellet was washed with 70% ethanol then dried and dissolved in RNase solution (0.5 x TE, 20 μg/mL RNaseA) and incubated at 37 °C for 30 min. Genomic DNA was purified using Genomic-tip (Qiagen, Germany) according to the manufacturer’s protocol.

### RNA extraction

Four-week-old leaves were sprayed with spores (10^6^ per mL) and incubated at 22 °C in 100% humidity in darkness. Leaves were harvested at time points of 16 h, and 1, 2, and 3 d after inoculation. Spores were sampled as a time point 0. Total RNA was prepared using PureLink Plant RNA Reagent (Thermo Fisher Scientific, USA), according to the manufacturer’s protocol. The RNA samples were treated with TURBO DNase (Thermo Fisher Scientific) to remove contamination from genomic DNA.

### Library preparation and sequencing

Libraries for paired-end reads and mate-pair reads of various insert sizes, including 2, 4, and 6 kbp, were constructed using the TruSeq DNA LT Sample Prep Kit and the Nextera Mate Pair Sample Prep Kit (both Illumina, USA), respectively. The paired-end library was sequenced on the Illumina MiSeq platform, while the mate-pair libraries were sequenced on the HiSeq 2500 platform (Illumina). For RNA-seq analysis, 4 μg total RNA was used to construct cDNA libraries using the TruSeq Stranded Total RNA Library Prep Kit (Illumina), according to the manufacturer’s instructions. The libraries were used for paired-end sequencing in 2 × 75 cycles on the NextSeq 500 platform (Illumina) in the high output mode. The sequencing reads were filtered for their Phred quality score, and reads with a quality score of ≥30, comprising ≥90% of the reads, were retained.

### Genome size estimation by k-mer distribution

Genome size was estimated by analyzing the k-mer frequency using the paired-end short reads. The peak of the k-mer frequency (M) of the reads is correlated with the real sequencing depth (N), read length (L), and k-mer length (K), and their relationships can be expressed by the following formula: M = N × (L – K + 1) /L [49]. The peak of the 15-mer frequency from the paired-end reads of *S. graminicola* was 37 (Fig. 2a). We divided the total sequence length (14,257,601,560 bp) by the real sequencing depth (39.398) to obtained an estimated the genome size of 361,885,068 bp (approx. 360 Mbp).

### Genome assemblies

All sequence reads in the FASTQ format were filtered for quality using the FASTX-Toolkit version 0.0.13 [50]. The paired-end reads from Miseq were processed by removing 10 bp of the 3′-end of the second reads, and then the first reads and the trimmed second reads with a Phred quality score of ≥20, comprising ≥80% of the reads, were retained. For mate-pair reads, only those sequence reads with a Phred quality score of ≥30, comprising ≥90% of the reads, were retained. Adaptor trimming and the removal of mate-pair reads with the wrong insert sizes were performed using an in-house pipeline of scripts written in Perl and C++. Finally, the paired-end and mate-pair reads were assembled using Platanus v.1.2.1 [18].

### Repeat element masking

Repeat elements were masked using RepeatModeler v1.0.8 [26]. RECON v1.08 [51] and RepeatScout v1.0.5 [52] were used to perform de novo repeat element prediction. Repbase library version 20,140,131 [53] was imported to RepeatModeler for reference-based repeat element searches. The final set of predicted repeat elements were then masked in the genome assembly using RepeatMasker v4.0.5 [27].

### Gene predictions

Genes were predicted based on ab initio and RNA-seq data. RNA-seq reads were assembled and mapped to the assembled genome using the Trinity/PASA pipeline. Redundant cDNA and protein sequences were merged using cd-hit and cd-hit-est., respectively, with a 90% sequence identity level. RNA-seq reads were also mapped to the assembled genome using the TopHat2/Cufflinks/PASA pipeline, and redundant cDNA and protein sequences were merged using cd-hit and cd-hit-est., respectively, with a 90% identity level. Predicted genes from Trinity/PASA and TopHat2/Cufflinks/PASA were merged, and redundant genes were merged with a 100% sequence identity level. The results were used as evidence for an expressed gene. A SNAP HMM, trained using the CEGMA output, and GeneMark-ES were used to generate sets of gene models. We ran MAKER2 [23] (first round) using these expressed genes, and the outputs from SNAP HMM, GeneMark-ES, and RepeatMasker. A SNAP HMM was then trained using the MAKER2 first-round output, and was used to re-run MAKER2. The intron-exon boundaries were predicted by AAT [20] using RepeatMasker output and the list of putative expressed genes. Finally, the results of the MAKER2 second round were merged with the evidence of gene expression and the AAT output using EvidenceModeler. Genes encoding complete protein sequences, whose expression was determined in the RNA-seq analysis, were defined as predicted genes.

### Phylogenetic analyses

Phylogenetic analyses were conducted using the orthologous genes predicted by CEGMA pipeline or annotated proteins using the Maximum Likelihood method implemented in MEGA6.06-mac [54], with 1000 bootstrap replicates.

### Orthology analyses

Orthology analyses were performed with the OMA [28] software, using a minimum score cut-off of 180 to define orthologous proteins among the five oomycete genomes. Genomic and protein sequences of *Plasmopara halstedii* [13] were obtained from their local server [http://dataportal-senckenberg.de/database/metacat/rsharma.26.4/bikf], and other oomycete species were obtained from Ensembl database [http://www.ensembl.org/index.html].

### TribeMCL analysis

Protein sequences of the putative secreted proteins from Sg and 11 oomycetes were clustered into families by TribeMCL algorithm [32] using BLASTp with an e-value cut-off of 1.0e-10. Protein sequences of 11 oomycetes were obtained from local server [https://www.dropbox.com/s/q37suzp15jkzshk/oomycetes_11species_secretomes.faa.zip?dl=0].

### Secreted protein predictions

Signal peptides were predicted using SignalP4.1 [29]. Mature proteins lacking signaling peptides were checked for transmembrane domains using TMHMM [55].

### Functional annotations

Functional annotations of predicted genes were added using InterProScan 5.15–54.0 [56] and the PANTHER classification system [57]. Protein family mapping was performed using pantherScore v.1.03, with the PANTHER database v11.

### Crinkler (CRN) protein predictions

First, CRN pre-candidates were identified by their sequence similarity to known CRN proteins using BLASTp. The resulting 12 proteins with a LF/YLAK motif in their N-terminal 120 amino acids (aa) were used in a manual HMM search. The HMM was trained from the N-terminal 120 aa of these genes, and the pre-candidates were searched using HMMER v3.1 [58] with an e-value cut-off of 1e-3. The resultant proteins were identified as CRN-like proteins.

### RXLR protein predictions

Candidate RXLR-like proteins were extracted from predicted secreted proteins using Perl regular expressions, HMM, and a BLASTp search. An initial set of proteins were searched using Perl regular expressions as described previously [33] and in HMM using the hmm profile [59]. The following approaches and criteria were used to extract exact RXLR proteins: (1) signal peptides within residues 1–30 followed by an RXLR motif [33, 59]; (2) Regex: allowing for a signal peptide between residues 10–40, followed by the RXLR motif within the next 100 residues, followed by the EER motif, allowing D and K [33]; (3) HMM search using Win’s hmm profile.

To complement the above approach, the predicted secreted proteins were scanned using HMM and a BLASTp search to extract RXLR-like proteins: (4) an HMM was trained on 40 aa sequences including the RXLR-EER motif from the exact RXLR proteins, and putative secreted proteins were searched for using HMMER v3.1 [58] with an e-value cut-off of 1e-3. (5) Putative secreted proteins with sequence similarity to known RXLR proteins were searched using BLASTp with an e-value cut-off of 1e-10.

The results for approaches 1–5 above were merged and the non-overlapping set of proteins were defined as RXLR-like protein genes (Additional file 18).

### WY-domain predictions

The WY-domains of predicted RXLR-like proteins were extracted using a pfam search, MEME [60], PSIPRED [61], and HMM, as described previously [38]. First, conserved motifs annotated as RXLR by the pfam search (Additional file 19) were searched using MEME with following parameters: -protein -oc. -nostatus -time 18,000 -maxsize 60,000 -mod zoops -nmotifs 5 -minw 6 -maxw 50. The protein secondary structure was predicted using PSIPRED (http://bioinf.cs.ucl.ac.uk/psipred/). From the MEME results, motif 1 included repeating WLY sequences and spanned an a-helical fold (Additional file 20: Fig. S5). We used sequences including motif 1 for the manual HMM search as a WY-domain. After training the HMM, the RXLR-like proteins were searched using HMMER v3.1 [58] with an e-value cut-off of 0.05 (Additional file 18).

### Expression profiling

Expression levels of predicted genes were determined using the TopHat2/Cufflinks pipeline [24, 25]. Differential expression was evaluated by the Fisher’s exact test using the edgeR package (version 3.18.1) [30]. TPM was calculated by the following formula: TPM = (FPKM / (sum of FPKM over all transcripts)) * 10^6^. Clustering by the ward’s method was performed using R Commander [62]. Clustering by logFC-Cosine method was performed using the cosine similarity of the vectors of their logFC values calculated by edgeR. Clustering by model-based clustering method was performed using MBCluster.Seq package (version 1.0) [31]. Expression levels of putative pathogenicity genes were indicated in Additional files 14, 15, 16, and 21.

### qRT-PCR analysis

cDNA was synthesized using ReverTra Ace® (Toyobo, Osaka, Japan). The qRT-PCR was performed using StepOne ™ real-time PCR instrument (Applied Biosystems, Foster city, CA, USA) with 10 μL reaction mixtures containing 0.5 μL cDNA, 5 μL the KAPA SYBR FAST Universal 2X qPCR Master Mix (Kapa Biosystems, Wilmington, MA, USA), 0.3 μL of each gene-specific primer (0.1 mM), and 1.9 μL ddH_2_O under the following reaction conditions: 95 °C for 20 s, followed by cycling for 40 cycles of denaturation at 95 °C for 3 s, and annealing and extension at 60 °C for 30 s. Finally, melt curve analyses (from 60 to 95 °C) were included at the end to ensure the consistency of the amplified products. A comparative CT (ΔΔCT) experiment used an endogenous control to determine the quantity of target in a sample relative to the quantity of target in a reference sample. Histone H2A gene (SG05345) was used as internal control. The primer sequences are provided in Additional file 22.

### Ploidy analysis

The ploidy level was estimated as described previously [63]. Paired-end reads were mapped to the assembled genome using BWA. SNPs with at least 10 × coverage were counted using samtools v0.1.18.

### Heterozygosity

To calculate heterozygosity, paired-end reads were mapped to the assembled genome using BWA. The SNPs were counted using samtools v0.1.18. SNPs with an allele frequency of between 0.4 and 0.6 were counted as heterozygous.

### Domain search for *S. italica* jacalin-like proteins

*S. italica* proteins were downloaded from the foxtail millet database of the Beijing Genome Initiative [64]. Jacalin-like domain-containing proteins were identified using InterProScan 5.15–54.0 [56] and the *S. italica* jacalin-like proteins were annotated using the HMMER web server [65].

## Additional files

Additional file 1: Table S1. BLASTn results of the assembled scaffolds against the nt NCBI database. (XLSX 54 kb)

Additional file 2: Table S2. Putative transposable elements in the *Sclerospora graminicola* genome sequence. (XLSX 51 kb)

Additional file 3: Table S3. Number of ortholog groups within the oomycete genomes. (XLSX 46 kb)

Additional file 4: Table S4. Gene IDs for nitrogen and sulfur assimilation enzymes in *Sclerospora graminicola* and related oomycetes. (XLSX 52 kb)

Additional file 5: Enrichment analysis of InterProScan domains between *Sclerospora graminicola* and related oomycetes. (XLSX 41 kb)

Additional file 6: Table S5. TPM values of DEGs encoding putative secreted proteins and cluster numbers from clustering analyses. (XLSX 68 kb)

Additional file 7: Figure S1. qRT-PCR analyses of differentially expression genes. (PDF 92 kb)

Additional file 8: Table S6.1 Summary of interproscan domain enrichment of DEGs encoding putative secreted proteins. Clusters I to IV correspond to the expression profiles given in Figure 3. **Table S6.2** Summary of interproscan domain enrichment of putative secreted proteins clustered using logFC-Cosine method. **Table S6.3** Summary of interproscan domain enrichment of putative secreted proteins clustered using MBCluster method. (XLSX 13 kb)

Additional file 9: Figure S2. Heat map showing the expression patterns of DEGs encoding putative secreted proteins. Genes were clustered by logFC-Cosine method. Line plots of the expression patterns of each gene cluster. L16H: *Sg*-inoculated leaves 16 h after inoculation; L1D, L2D, and L3D: *Sg*-inoculated leaves at one, two, and three days after inoculation, respectively. (PDF 204 kb)

Additional file 10: Figure S3. Heat map showing the expression patterns of all genes. Genes were clustered by model-based clustering method. Line plots of the expression patterns of each gene cluster. SPO: mixture of sporangia and zoospores; L16H: SPO-inoculated leaves 16 h after inoculation; L1D, L2D, and L3D: SPO-inoculated leaves at one, two, and three days after inoculation, respectively. (PDF 124 kb)

Additional file 11: Summary of clustering results of TribeMCL. (XLSX 2667 kb)

Additional file 12: Figure S4. Distribution of gene expression values. Box plot of TPM of putative secreted protein genes (A) and genes clustered in *Sg*-specific Tribe of jacalin-like domain-containing proteins by TribeMCL. (B) (PDF 444 kb)

Additional file 13: Jacalin-like domain protein genes predicted in genome of *Sclerospora graminicola* and their expression levels during infection. (XLSX 49 kb)

Additional file 14: NLP genes predicted in genome of *Sclerospora graminicola* and their expression levels during infection. (XLSX 44 kb)

Additional file 15: CRN genes predicted in genome of *Sclerospora graminicola* and their expression levels during infection. (XLSX 43 kb)

Additional file 16: RXLR-like genes predicted in genome of *Sclerospora graminicola* and their expression levels during infection. (XLSX 82 kb)

Additional file 17: Jacalin-like domain containing protein genes of *Setaria italica.* (XLSX 58 kb)

Additional file 18: Candidate RXLR-like effectors of *Sclerospora graminicola*, predicted based on gene models. (XLSX 68 kb)

Additional file 19: Conserved motifs of *Sclerospora graminicola* protein sequences annotated as RXLR by the pfam search. (TXT 5 kb)

Additional file 20: Figure S5. Prediction of WY-motifs in SgRXLR-like proteins. (PDF 162 kb)

Additional file 21: Putative effector-like protein genes in *Sclerospora graminicola* and their expression during infection. (XLSX 46 kb)

Additional file 22: Primers sequences for qRT-PCR. (XLSX 56 kb)

## Abbreviations

CEGMA: Core eukaryotic gene mapping approach
CEGs: Core eukaryotic genes
CRN: Crinkler
CTAB: Hexadecyltrimethylammonium bromide
DM: Downy mildew pathogens
FPKM: Fragments per kilobase of transcript per million mapped reads
HMM: Hidden markov model
Kbp: Kilobase pair
LINE: Long interspersed elements
LTR: Long terminal repeat
Mbp: Megabase pair
NCBI: National center for biotechnology information
NLPs: Necrosis and ethylene-inducing peptide 1 (Nep1)-like proteins
NLR: Nucleotide-binding leucine-rich repeat
OMA: Orthologous MAtrix
SCRs: PcF-like small cysteine-rich proteins
SINE: Short interspersed nuclear element;
SNP: Single Nucleotide Polymorphism
