# Supplementary material for: Genome analysis of the foxtail millet pathogen Sclerospora graminicola reveals the complex effector repertoire of graminicolous downy mildews
Source: BMC Genomics. 2017 Nov 22;18:897. doi: 10.1186/s12864-017-4296-z (PMC5700566; doi:10.1186/s12864-017-4296-z)
Supplement: Supplementary file 7 — qRT-PCR analyses of differentially expression genes. (PDF 92 kb) [file 12864_2017_4296_MOESM7_ESM.pdf]

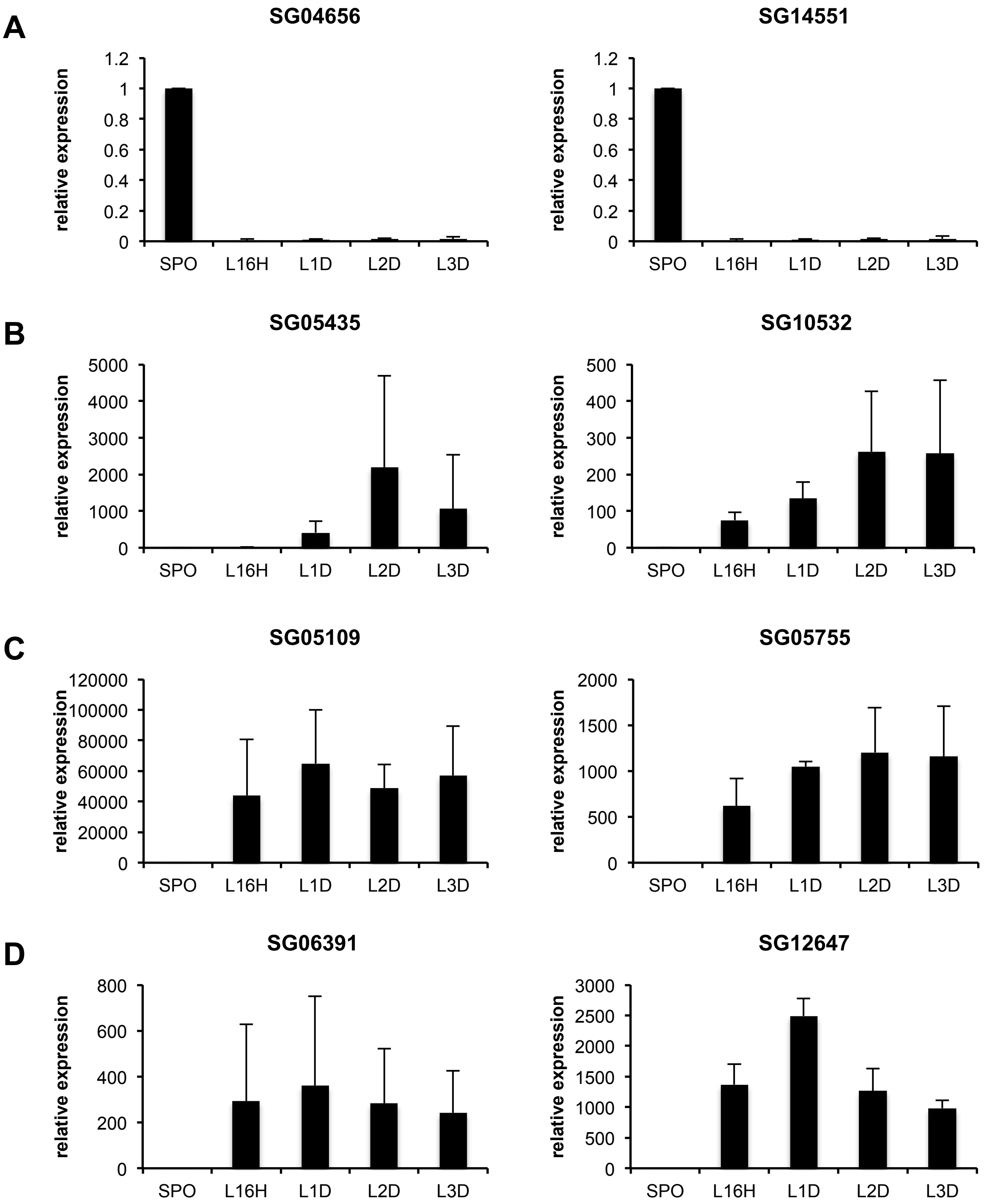


**Supplementary figure 1.** qRT-PCR analyses of DEGs of cluster I (A), II (B), III, (C), and IV (D). L16H: Sg-inoculated leaves 16 hours after inoculation; L1D, L2D, and L3D: Sg-inoculated leaves at one, two, and three days after inoculation, respectively. Cluster numbers are defined in Figure 3.
