# Supplementary material for: Genome analysis of the foxtail millet pathogen Sclerospora graminicola reveals the complex effector repertoire of graminicolous downy mildews
Source: BMC Genomics. 2017 Nov 22;18:897. doi: 10.1186/s12864-017-4296-z (PMC5700566; doi:10.1186/s12864-017-4296-z)
Supplement: Supplementary file 9 — Heat map showing the expression patterns of DEGs encoding putative secreted proteins. Genes were clustered by logFC-Cosine method. Line plots of the expression patterns of each gene cluster. L16H: Sg-inoculated leaves 16 h after inoculation; L1D, L2D, and L3D: Sg-inoculated leaves at one, two, and three days after inoculation, respectively. (PDF 204 kb) [file 12864_2017_4296_MOESM9_ESM.pdf]

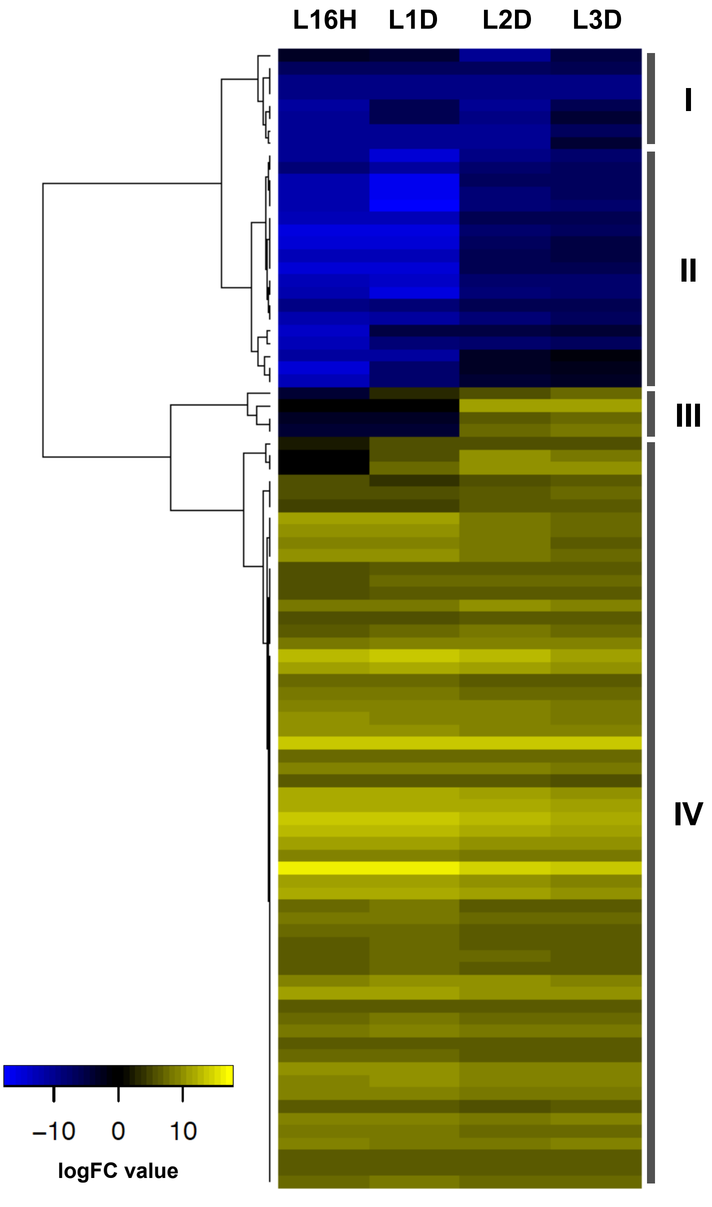

Supplementary figure 2. Heat map showing the expression patterns of DEGs encoding putative secreted proteins. Genes were clustered by logFC-Cosine method. Line plots of the expression patterns of each gene cluster. L16H: Sg-inoculated leaves 16 hours after inoculation; L1D, L2D, and L3D: Sg-inoculated leaves at one, two, and three days after inoculation, respectively.
