# Supplementary material for: Genome analysis of the foxtail millet pathogen Sclerospora graminicola reveals the complex effector repertoire of graminicolous downy mildews
Source: BMC Genomics. 2017 Nov 22;18:897. doi: 10.1186/s12864-017-4296-z (PMC5700566; doi:10.1186/s12864-017-4296-z)
Supplement: Supplementary file 12 — Distribution of gene expression values. Box plot of TPM of putative secreted protein genes (A) and genes clustered in Sg-specific Tribe of jacalin-like domain-containing proteins by TribeMCL. (B) (PDF 444 kb) [file 12864_2017_4296_MOESM12_ESM.pdf]

A **putative secreted protein genes**

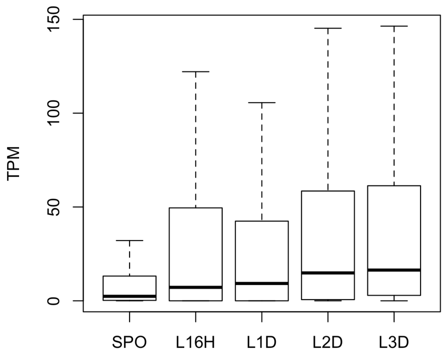

	SPO	L16D	L1D	L2D	L3D
Min.	0.0	0.0	0.0	0.0	0.0
1st Qu.	0.2	0.0	0.0	0.6	2.9
Median	2.4	7.2	9.2	14.9	16.4
Mean	101.7	144.2	146.0	134.2	123.5
3rd Qu.	13.2	49.5	42.5	58.5	61.3
Max.	27101.4	16331.4	22292.6	23446.9	19724.5

B

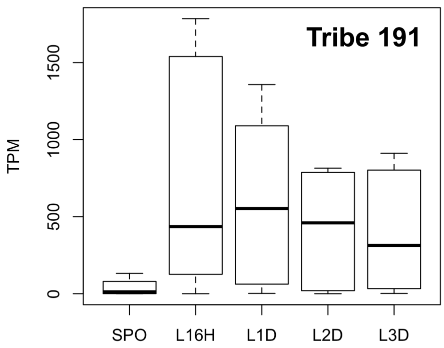

	SPO	L16D	L1D	L2D	L3D
Min.	0.0	0.0	2.1	0.0	1.9
1st Qu.	0.5	126.0	62.2	19.8	33.2
Median	12.2	435.8	553.1	459.8	314.0
Mean	371.6	2942.6	3414.0	3430.6	3122.8
3rd Qu.	80.0	1539.5	1090.0	787.9	802.5
Max.	3225.9	16331.4	22292.6	23446.9	19724.5

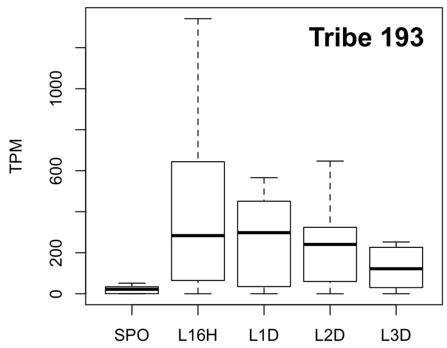

	SPO	L16D	L1D	L2D	L3D
Min.	0.0	0.0	0.0	0.0	0.0
1st Qu.	0.4	64.7	34.8	59.8	29.9
Median	21.5	283.5	297.9	240.3	122.0
Mean	19.1	396.3	330.3	220.9	122.1
3rd Qu.	34.0	643.7	451.1	323.9	226.1
Max.	51.5	1341.4	1224.9	646.9	252.4

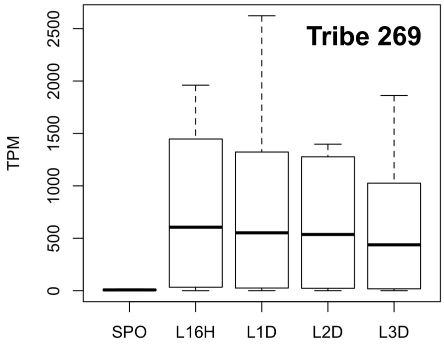

	SPO	L16D	L1D	L2D	L3D
Min.	0.0	0.0	0.0	0.0	0.0
1st Qu.	1.8	45.8	33.7	34.2	25.1
Median	7.2	605.5	552.1	536.2	437.6
Mean	10.4	766.4	802.8	633.7	603.0
3rd Qu.	12.8	1408.2	1294.9	1234.9	956.6
Max.	37.7	1960.5	2622.8	1397.6	1861.9

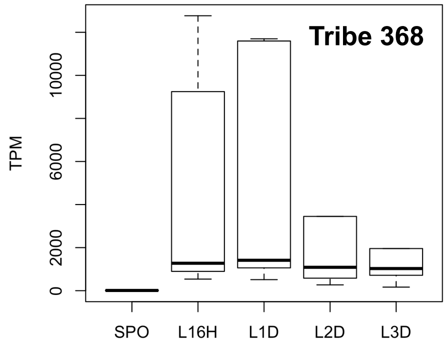

	SPO	L16D	L1D	L2D	L3D
Min.	0.0	538.0	512.8	270.2	165.8
1st Qu.	1.1	969.6	1099.2	646.8	753.8
Median	10.7	1274.1	1412.4	1086.2	1027.9
Mean	106.9	4332.8	4615.3	2783.7	2231.3
3rd Qu.	21.5	7273.8	9099.2	2919.1	1764.4
Max.	596.9	12768.6	11697.9	10229.1	8494.1

Supplementary figure 4. Distribution of gene expression values. Box plot of TPM of putative secreted protein genes (A) and genes clustered in *Sg*-specific Tribe of jacalin-like domain-containing proteins by TribeMCL (B).
