# Supplementary material for: Genome analysis of the foxtail millet pathogen Sclerospora graminicola reveals the complex effector repertoire of graminicolous downy mildews
Source: BMC Genomics. 2017 Nov 22;18:897. doi: 10.1186/s12864-017-4296-z (PMC5700566; doi:10.1186/s12864-017-4296-z)
Supplement: Supplementary file 20 — Prediction of WY-motifs in SgRXLR-like proteins. (PDF 162 kb) [file 12864_2017_4296_MOESM20_ESM.pdf]

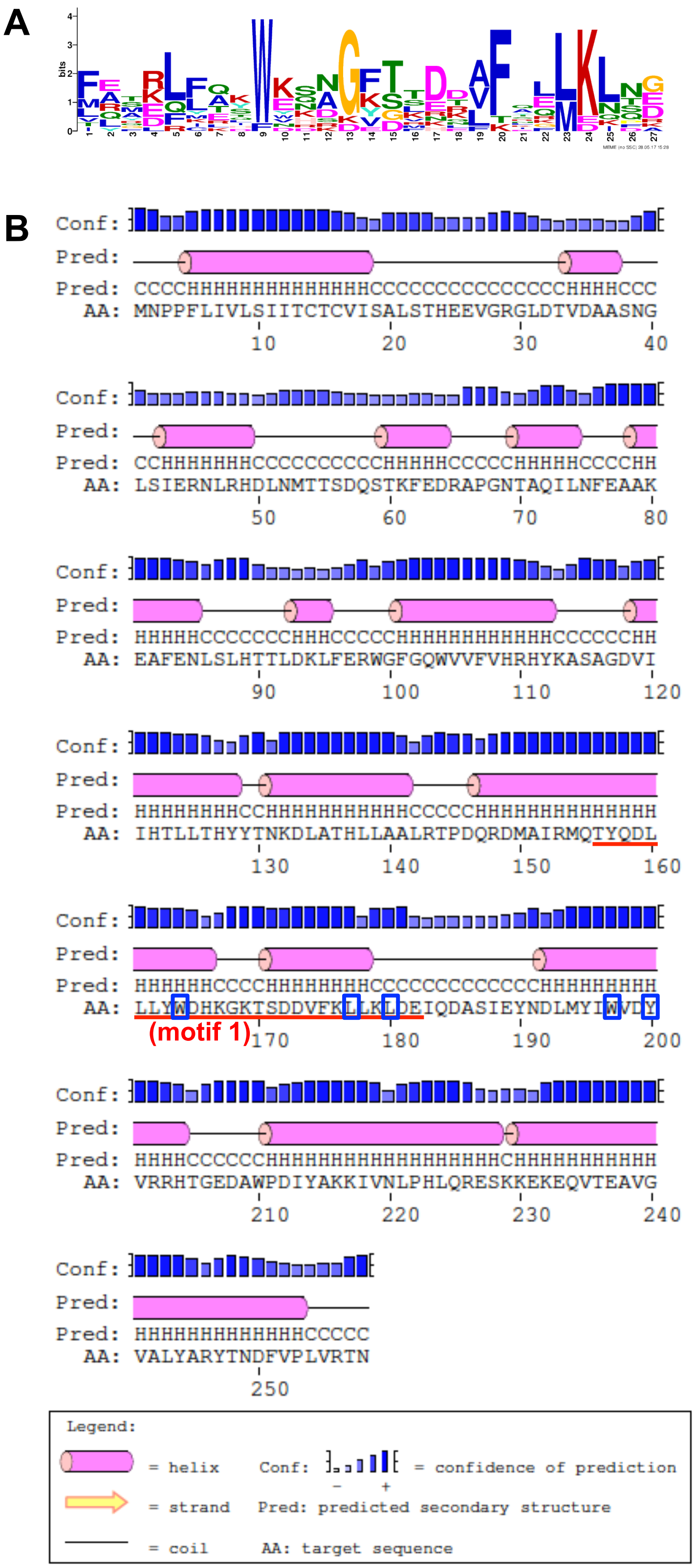


**Supplementary figure 5.** Prediction of WY-motifs in SgRXLR-like proteins. (A) Sequence motif (motif 1) identified by MEME. (B) Predicted secondary structure of the SG01003 protein by PSIPRED. Motif 1 is underlined in red. WLY-motifs are indicated by blue boxes.
